# Supplementary material for: Referral pattern of oral and maxillofacial surgery cases in Sudan: A retrospective age-and sex-specific analysis of 3,478 patients over four years
Source: PLoS One. 2021 Mar 29;16(3):e0249140. doi: 10.1371/journal.pone.0249140 (PMC8007037; doi:10.1371/journal.pone.0249140)
Supplement: Attachment — Submitted filename: response to reviewer.docx [file pone.0249140.s002.docx]

14 February 2021

Amit Sapra
Academic Editor

Dear editor:

Thank you for your favorable replay. I revised and submitted the manuscript with required changes to meet the journal style r, as well as dataset uploaded as Supporting Information files.

Sincerely,

Musadak A. Osman

Corresponding authors
